# Oral Electricity

**Published:** 1878-07

**Authors:** J. S. Cassidy


### THE DENTAL REGISTER.

VOL. XXXII.] JULY, 1878. No. 7.

### ORAL ELECTRICITY.

BY J. S. CASSIDY, D. D. S.

*Read before the Kentucky State Dental Society.*

MR. PRESIDENT AND GENTLEMEN:—Whenever a needed avocation or science depends for its development upon practical experiment, it must necessarily progress more or less rapidly toward the perfect consummation of the objective ideas which called it into existence. In our specialty the leading objective idea is the arrest of decay in the carious teeth of our kind, and thus far we must acknowledge that our idea has only reached the chrysalis period of its development, inasmuch as the many instances of unexpected failures in our experiments, (nearly all our operations are experimental), are, I fear, scarcely balanced by the instances of entire and permanent success.

If considered in connection with the vicissitudes constantly affecting the dental portion of the alimentary canal, the idea of removing the debris and frailties from a carious tooth, and then sealing the cavity with a substance, possessing, with other necessary attributes, the property of resisting mechanical and chemical abrasion, is the correct one, indeed the only one that could be, or perhaps will be generally adopted to arrest the disease.

Of the different preparations employed for filling teeth, gold in its various forms has been considered individually the most desirable; being endowed with certain physical and chemical properties heretofore conceived to be essential, such as fair adaptability, hardness, indestructability and a relatively not objectionable color, and although the open and illimitable field of science and art has thus far furnished conservative dentistry with but few available weapons to combat the insidiously persistent propagation of dental caries, there have been so many cases of evident success under real difficulties as to merit the enthusiasm of its disciples and the full confidence of the intelligent laity. In the light of experience and observation it is perfectly safe to assume that the careful use of gold in filling teeth has been the principal factor in producing and retaining this confidence in the ability of our profession to do good. This is the conscientious opinion of a large majority of respectable dentists, and were they restricted to the sole employment of any one material, gold would certainly be the favorite.

Now I do not wish to be understood as starting out an advocate of the mono-metallic theory, or any other monomania, "that a tooth which can be filled at all should be filled with gold." Such an aphorism is not according to my notions of accepted dentistry, for our profession is progressive and therefore truly eclectic in the full meaning of the term. I wish merely to place myself on the side of truth, and to protest against the dictum of the so called "new departure," that a gold filling in all cases is a delusion and a snare.

At a meeting of the American Dental Association held in 1875, my friend, Dr, S. B. Palmer, of Syracuse, introduced in the report on chemistry a statement of conclusions he had arrived at by experiments, on the subject of oral electricity. Let me quote briefly:

"There are two distinct classes of teeth which come under our care. The first are those which are well organized or in a normal condition, except that they have become decayed and demand our attention. For such I recommend the same as others, viz: gold; because there is nothing objectionable in the material, and it is a purely mechanical operation, filling the cavity, excluding the moisture, and checking the chemical action, The other class of teeth are of a chalky, undeveloped character, which require to be treated differently. Examples of this class in the six year old molars of children. In the cavities of such teeth I find that gold is not the best preservative agent; there is elctro-chemical decomposition of the tooth substance induced by an electric current set up between the dentine and the gold, because in a porous, poorly organized tooth there is moisture allowing circulation through the pores of the dentine. Well organized dentine is a non-conductor, insert the gold, make a moisture tight filling and the tooth is preserved indefinitely."

According to this statement, Dr. Palmer considered solution or fluidity an indispensable precedent, condition to the galvanic process; but soon a few others took up the line of experiments, and in a little while Dr. Palmer's honestly acquired prestige could be seen only "through a glass darkly," for they at once appeared to desire the utter extermination of gold. This theory merely explained the reasons for exceptional failures, while the "new departure" (consisting mainly of Drs. Chase, of St. Louis, and Flagg, of Philadelphia) must introduce theories apparently founded on incontrovertible expeiiment to prove that which stubborn experience denies; that gold, even in the favorable cases of Dr. Palmer's first class of strongly developed teeth, invites destruction by its presence, though the most perfect union exist between it and the tooth, vide Dr. Chase: "Every tooth containing a plug is a galvanic battery, and is put into action the moment a portion of acidulous fluid touches both plug and dentos. If the plug is water tight then the action is limited to the margin; if it be a leaky one, then in addition to the points of marginal contact there is action inside of the cavity at every point where the material of the plug and dentos are in contact, and *such a battery once started proceeds continuously*," its force being greater between gold and dentine than between the latter and any other material used for filling. Could any theory be more destructive to every principle of observed and accepted facts than this? The best means which nature and art have permitted us to use against decay, are worse than failures, and dentistry, than which no other calling proportional to the number of its disciples, has conferred so much of comfort upon man, for fifty years has been a diabolical fraud.

> "But now when time has made the imposture plain,
> What new delusion charms your cheated eyes again?"

Absolutely nothing; they offer us alas! only those things we already knew, for "lo! these many days."

"If the plug is water tight then the action is limited to the margin of the plug and dentos." That is, the positive element of the battery will be attacked only at the line of perfect union with the negative. It means that or nothing. Now numerous experiments do not prove this assertion, but on the contrary, that when a tooth filled or not filled is submerged in a dilute bone dissolving acid, at a temperature of 90°F. the entire surface becomes abraded, the loss varying in depth according to the resistance offered by the different regions of enamel and cementum. Teeth are never primarily affected by acids taken into the mouth, except by general surface decomposition; the destroying agents are *developed* there, and do their mischievous work at the immediate point and instant of their formation. A well marked acid condition of the human saliva is extremely rare, but even though it did exist as often as one would be led to suppose by the flippant stereotyped expression, "acid fluids of the mouth," the results of such a condition would be solution manifested on those parts least able to resist the affinity of the acid. If the dentine be exposed, either by recent decay, or by imperfect sealing of a cavity, by filling with gold, amalgam or other material, it will suffer in proportion to its weakness, independent of electric action. If the condition of the filling be "water tight," the points of "marginal contact" will be as free from attack as any other equally impervious portion of the tooth. And right here hangs the whole practical question, does such a filling primarily assist in inducing the production of an acid from the saliva? I can not reconcile the little that is known of electricity with such a proposition. Normal saliva contains no ingredient capable of reacting directly on osseous matter. Sodium chloride, the principal salivary electric excitant, is perfectly neutral to dentine; it does not attack the latter in the least, and in order to induce galvanic action in such a case, its halogen, (or with other electrolytes, the negative radicle), must be able to combine directly with the positive constituent of the supposed cell.

Antecedent production of a solvent of the positive element is a necessity to the galvanic process. One example of the numerous methods of such production will suffice. The first stages of fermentation of extraneous matter in saliva often induces the decomposition of sodium chloride in order to supply chlorine to nascent hydrogen, but the molecules of hydrochloric acid thus obtained would attack the dentine or enamel at the point of their formation or not at all. If developed in juxtaposition to a filling of any kind, then solution would take place, regulated in amount by the exercise of affinity between the chlorine of the acid and the molecules receiving the attack. The oppositeness of the polarities existing between the two solids—say a gold filling and dentine—would certainly be increased at the point of closest contact, but never to the extent of adding to the positive osseous matter, an extra attractive power for the molecules of negative chlorine, sufficient to convey the latter to the part of greatest polarization, viz: the point of union between the gold and dentine or enamel; which position, according to the electric theory, the molecules must grasp or remain inert, whilst in practice we know that those positions are the very last to succumb. That is the idea in a nutshell, the latest phase of the "new departure," and the one too not corroborated by indisputably scientific experiments, such as are described by its advocates.

The mere deflection of the needle of the galvanometer does not prove that a solid is being dissolved. Difference in temperature, or difference in the conducting power of the parts experimented with, when bathed in a liquid containing matter undergoing any kind of chemical transformation, (such as is always present in saliva), would disturb the electric equilibrium, as manifested by the needle, in equal ratio to the chemical excitement occasioned by solution of a solid. "Facts are stubborn things," and facts are just as stubborn in opposing so called science as in any other condition or circumstance.

But while I can not agree with the proposition that mere oppositeness of polarity between gold and dentine produces *per se*, and localizes concomitant chemical action, I readily coincide with Dr. Palmer in that there are classes of teeth, and also special conditions wherein gold is inadmissible. The difficulties in these cases, however, are *a priori* purely physical. The tooth substance is wholly or in part too soft or porous and sometimes too frail to receive the sudden pressure or blow necessary to condensation: it suffers contusion and if too frail then fracture; or the location of the cavity is such as to render perfect manipulation of the gold impossible. A poorly sealed cavity in either case is the consequence. Such a condition suggests further decay, but not by means of galvanic currents generating from the interposing liquid the necessary solvent. The latter agent must first be formed by independent recomposition of elemental or compound radicles, set free from the fermenting liquid. The power of dissolving presupposes the ability of the agent to primarily attack the tooth, and then only and for the time being is galvanic action established to the extent of the existing polarities of the gold and dentine. Opposite polarity may increase the energy of the solvent, or rather weaken the resistance of the positive substance just so far as the conductivity of the latter can influence the attraction, but the most unfortunately favored dentine in this regard can not convey the induced excitement beyond the immediate neighborhood of its polarized opponent, hence solution of continuity, reaching widely from the negative filling, instead of hugging the points of marginal contact where the greatest polarity exists, can not be supposed to be influenced by electric currents beyond an infinitely narrow sphere. Furthermore, I can not understand how galvanic action once established between a filling and dentine remains continuously. It continues, (limited in extent by polarity), only so long as the fluid furnishes the local solvent.

Now aside from any electric incompatibility, the advantages of plastic fillings reside in the ease by which they are perfectly adapted to inaccessible cavities, softness of tissue or frail walls, securing thus for the time being the most complete exclusion from the dentine of all destroying agents, but if subsequent solution of enamel or cementum occurs, decay proceeds as surely, and (other conditions being equal), I doubt not as readily as though gold were present.

Plasticity, however, is the great desideratum which future dentistry must secure in nonmetallic compounds for filling teeth. They may, if need be, be reasonably expensive, and require a high order of skill to manipulate, they must be infinitely superior in the property of indestructibility to the mere makeshifts of to-day, and perfectly equal with adjoining enamel in reflecting the same rays of light. Then, and only then, will the refined operations in gold become things of the past.

